# Pulsed field ablation for atrial fibrillation: a comprehensive bibliometric analysis of research trends and emerging Frontiers

**DOI:** 10.3389/fcvm.2025.1513942

**Published:** 2025-02-27

**Authors:** Li Li, Bin Xie

**Affiliations:** ^1^ Department of Internal Medicine, Chaozhou Central Hospital, Chaozhou, Guangdong, China; ^2^ Department of Cardiovascular, The Second Affiliated Hospital of Shantou University Medical College, Shantou, Guangdong, China

**Keywords:** atrial fibrillation, pulsed field ablation, bibliometric analysis, cardiac electrophysiology, ablation techniques

## Abstract

**Background:**

Atrial fibrillation (AF) is the most prevalent cardiac arrhythmia worldwide, posing significant health burdens. Pulsed field ablation (PFA) is an emerging non-thermal technique that is gaining traction due to the ability to selectively target myocardial cells and minimize damage to surrounding tissues. We conducted a comprehensive bibliometric analysis of PFA use in AF treatment to map research trends, collaborations, and future directions.

**Methods:**

We extracted data from the Web of Science Core Collection on September 6, 2024, using search terms related to PFA and AF. Publication trends, citation trajectories, collaborative networks, and keyword co-occurrences were analyzed utilizing tools such as Bibliometrix R, VOSviewer, and CiteSpace.

**Results:**

In total, 217 publications were retrieved. The number of publications increased rapidly from 2019 to 2024, with a notable surge occurring after 2022. Contributions from the United States, Germany, and China accounted for more than 60% of all publications. The institution with the largest output was The Icahn School of Medicine at Mount Sinai. The most productive journals were *Europace* and the *Journal of Interventional Cardiac Electrophysiology*. Prolific authors were identified, underscoring significant international collaborations. The most cited publications highlighted the efficacy and safety of PFA. Keywords with strong recent citation bursts included “tissue”, “cardiomyopathy”, and “closed chest ablation”.

**Conclusion:**

PFA is becoming established as a viable alternative for AF ablation, showing promising safety and efficacy. This bibliometric analysis confirmed the growing scientific interest and collaborative efforts in this field, suggesting that robust future developments will occur.

## Introduction

Atrial fibrillation (AF) is the most common persistent cardiac arrhythmia worldwide (1), affecting millions and contributing significantly to the global burden of heart failure and stroke (2, 3). Despite the availability of various treatments, catheter ablation remains a cornerstone in AF management (4). However, traditional techniques such as radiofrequency ablation (RFA) and cryoablation have limitations, including high recurrence rates and complications (5, 6). Pulsed field ablation (PFA), an emerging non-thermal ablation technique, has garnered increasing attention due to the ability to selectively target myocardial cells while sparing surrounding tissues, potentially revolutionizing AF treatment (7, 8).

In recent years, the body of research on the use of PFA for AF has grown substantially. Recent studies have demonstrated significant advantages of PFA in terms of safety and efficacy, particularly the potential to reduce collateral damage to critical structures such as the esophagus and blood vessels due to its non-thermal mechanism (9–11). However, despite the promising results obtained with the use of this innovative technology, a comprehensive analysis of PFA research trends and emerging areas remains lacking.

Bibliometric analysis is a powerful tool for the elucidation of research trends and identification of emerging frontiers in a specific field (12). The quantitative assessment of the publication volume, citation impact, and research hotspots provides a clearer picture of the scholarly landscape (13). Such analysis is critical for the highlighting of key areas of development, identification of gaps in the literature, and guidance of future research directions (14). To date, no systematic bibliometric analysis of the literature on PFA use in the context of AF treatment has been conducted, underscoring the need to map out research trends, collaborative networks, and future opportunities in this rapidly advancing field.

In this study, a comprehensive bibliometric analysis of research on the use of PFA in the treatment of AF was conducted. We explored publication trends, citation trajectories, networks of collaboration among leading researchers and institutions, and the evolution of key research themes. Our findings offer valuable insights into the future development of PFA application in AF treatment and contribute to the optimization of this emerging technology on a global scale.

## Methods

### Data source and search strategy

This study was conducted with data from the Web of Science Core Collection (WoSCC) (15), including the Science Citation Index and Social Sciences Citation Index databases. The search was performed using the following terms: topic search (TS) = (“pulsed field ablation” OR “pulsed electric field ablation” OR “pulsed field” OR “PFA”) AND TS = (“atrial fibrillation” OR “AF” OR “pulmonary vein isolation” OR “PVI”). We limited the search to articles and reviews published in English to ensure data consistency and reliability. Data were extracted on September 6, 2024, to include the most recent publications.

### Data analysis and visualization

For the comprehensive bibliometric analysis, we utilized several software tools. The Bibliometrix R package (version 4.3.0) (16) was used primarily for quantitative analyses, such as the evaluation of the strength of collaborations between countries. This tool was also used to create three-field plots visualizing the relationships and interactions among institutions, keywords, and countries.

VOSviewer (version 1.6.20) (17) was employed to calculate publication and citation counts, keyword frequencies, and co-authorship and co-occurrence, with the construction and visualization of networks of collaboration among authors and institutions and the mapping of keyword co-occurrences. Its built-in clustering algorithms were used to identify key themes and research hotspots in the scientific literature. Additionally, VOSviewer's time-overlay functionality facilitated the dynamic visualization of network development and evolution over different periods.

CiteSpace (version 6.3.R1) (18) was used for citation analysis and visualization to identify highly cited publications and keywords with significant citation growth during specific periods. This “scientific knowledge mapping” approach provided an intuitive understanding of research frontiers and highly influential academic contributions.

## Results

### Annual publication trends

**Figure 1:**
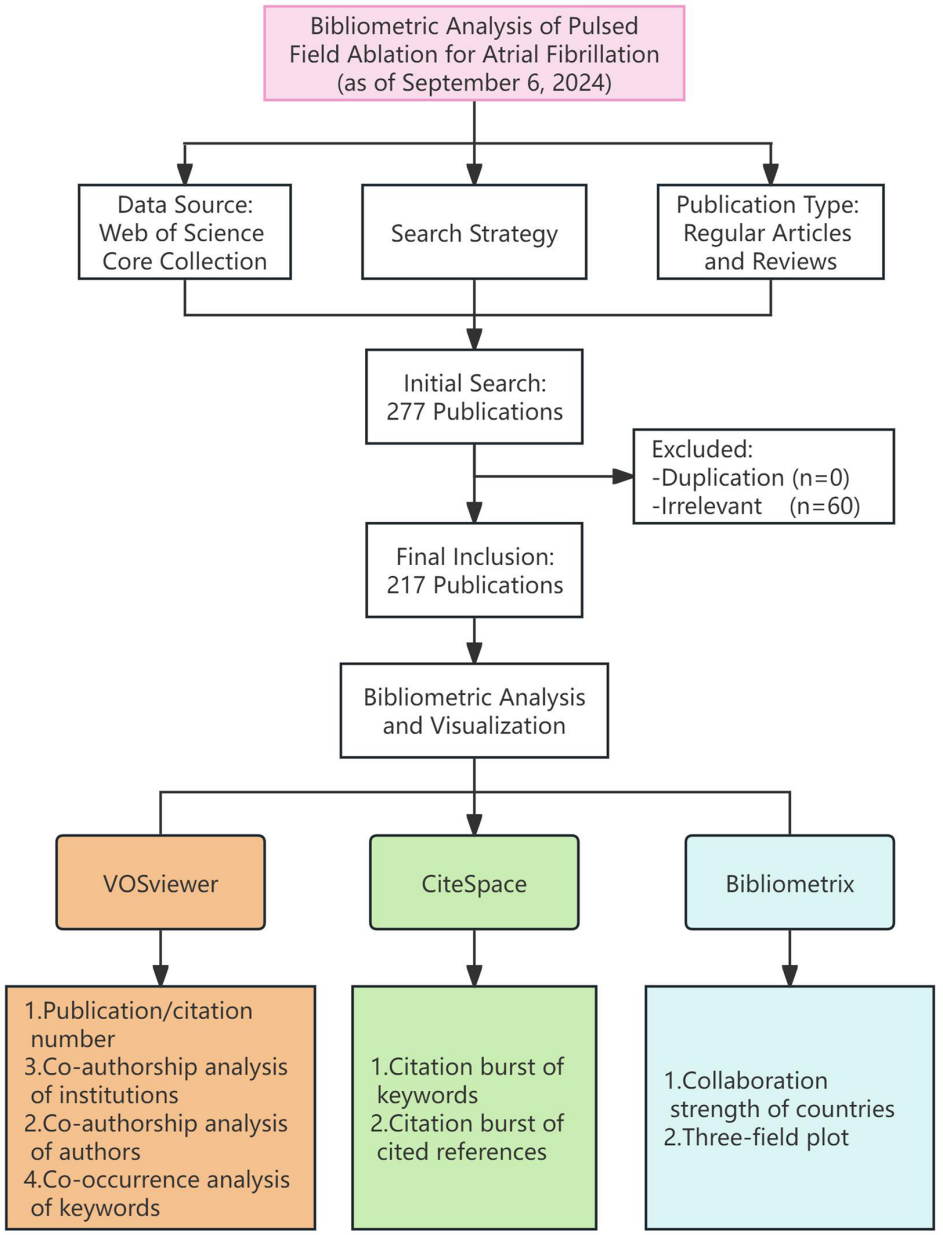
Flow of the study.

**Figure 2:**
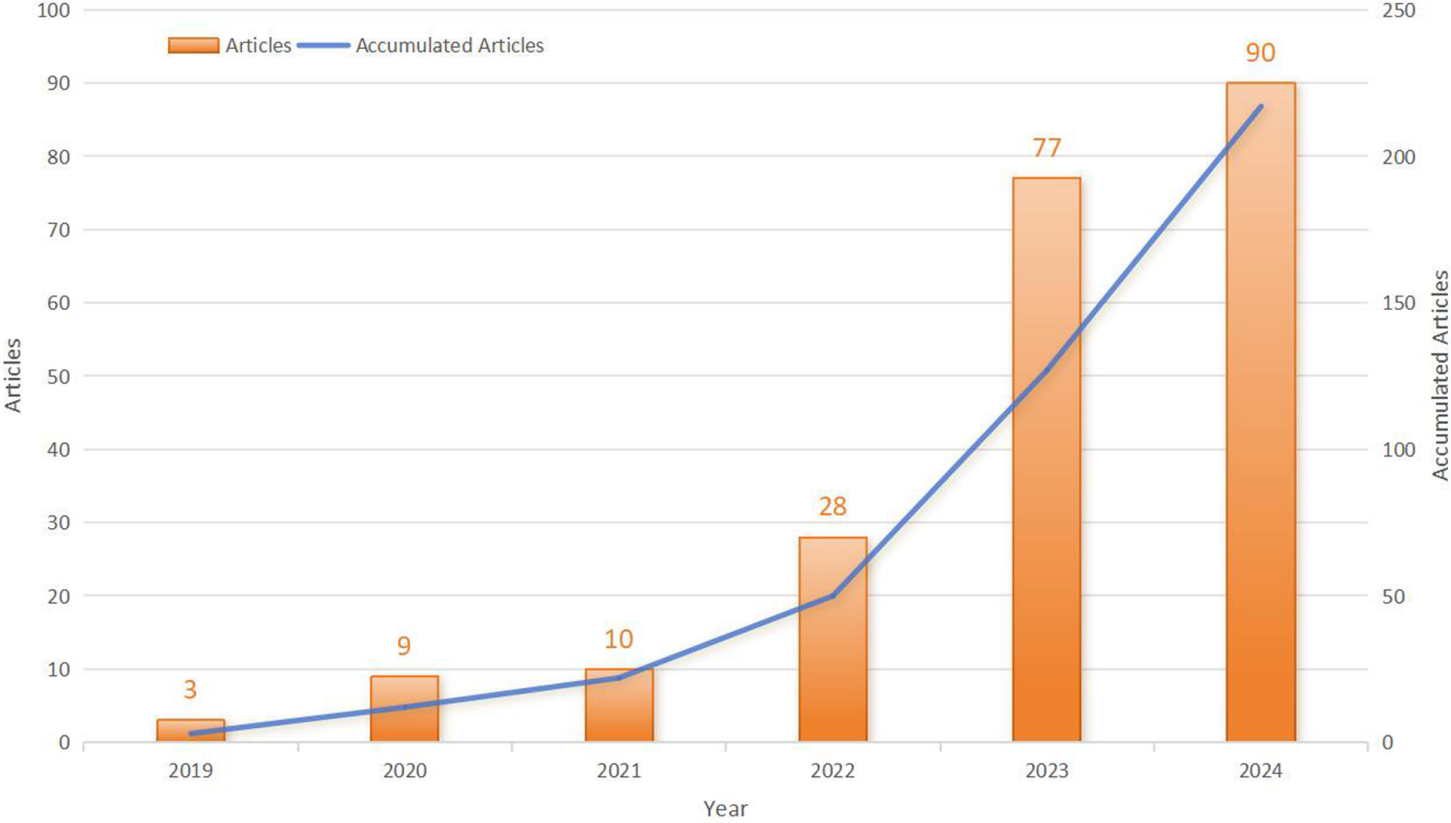
Numbers of publications per year and cumulatively.

Initially, 277 publications related to the use of PFA in AF treatment were retrieved. After the exclusion of 60 duplicate and irrelevant studies, 217 publications remained (Figure 1). Of these publications, 80.6% were original research articles and 19.4% were review articles. Between 2019 and 2021, research on the use of PFA for AF was in its nascent stage, with a modest but steadily increasing output (from 3 articles in 2019 to 10 articles in 2021). The field expanded rapidly after 2022, with the number of publications soaring from 28 in 2022 to 90 in 2024, constituting an impressive average annual growth rate of 97.44% (Figure 2).

### National publication counts

**Figure 3:**
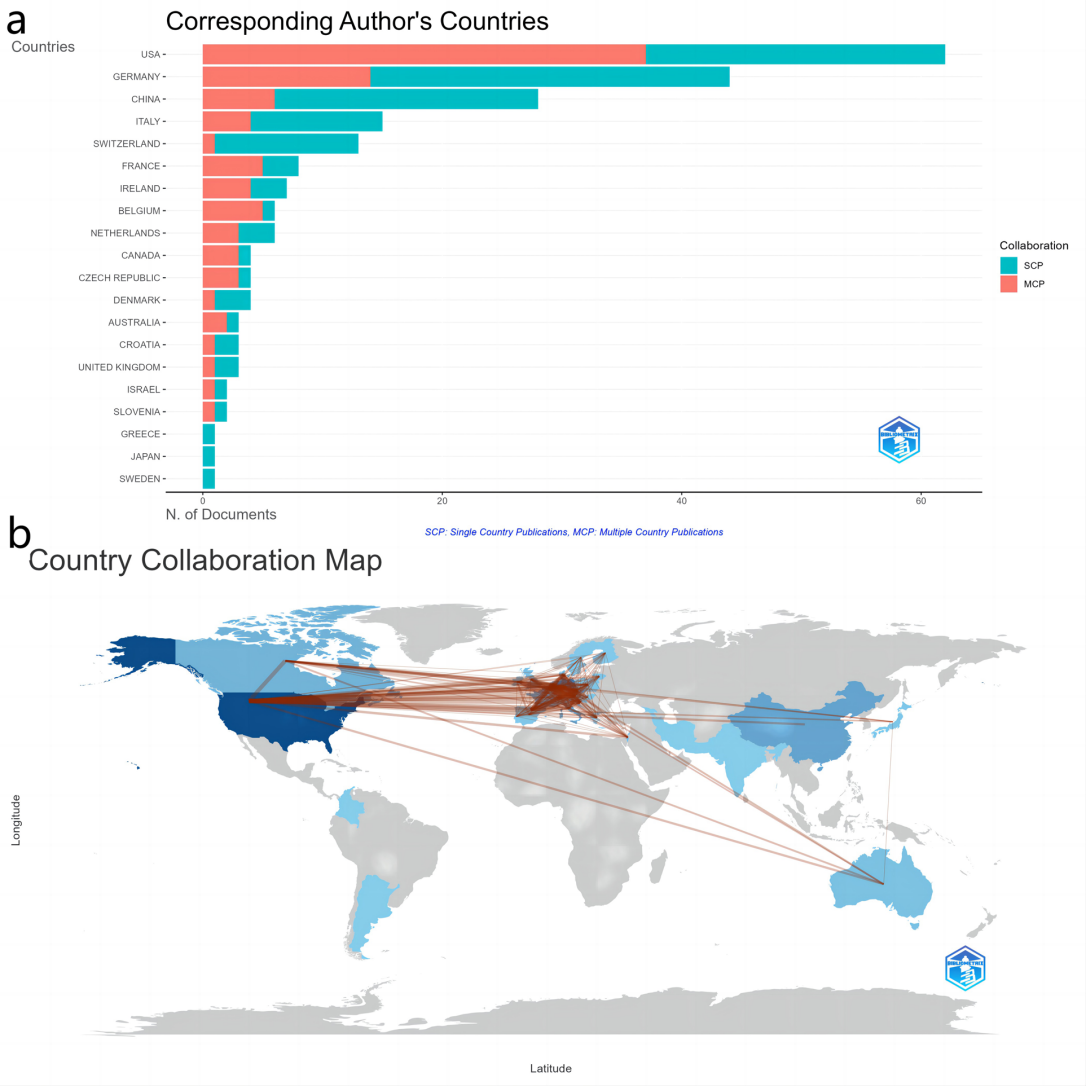
Global contributions and collaborations in research output. **(a)** Top 20 countries by article output. **(b)** Map of international collaboration.

Thirty-six countries and regions have contributed articles in this field. The United States leads, with 62 articles accounting for 28.57% of the total publications, followed by Germany with 44 articles (20.27%) and China with 28 articles (12.9%). Collectively, scholarly work from these three nations accounts for more than 60% of the total, highlighting these countries' pivotal roles in the advancement of PFA research (Figure 3A). The remaining countries in the top 10 list, particularly those in Europe, have still exerted significant influence despite having fewer publications, demonstrating robust research activity.

Figure 3B shows the network of collaboration among these countries and regions. The United States is at the center of international cooperation, contributing authors to 37 multi-country publications, which highlights its strong bilateral ties with various nations. The most prolific collaboration has been between the United States and the Czech Republic, with 31 joint publications, showcasing a robust scientific partnership (Supplementary Table S1). Additionally, the United States has maintained notable collaborations with France (19 publications), Belgium (15), Canada (15), and Germany (14), further emphasizing its central role not only in publication output but also in the fostering of global research networks. Among the top 10 collaborations, those between European countries such as France and the Czech Republic (15 joint publications), and Germany and the Netherlands (14 joint publications), display active engagement, underscoring deep connections in work on PFA use for AF in the European research community.

### Institutional publications

**Figure 4:**
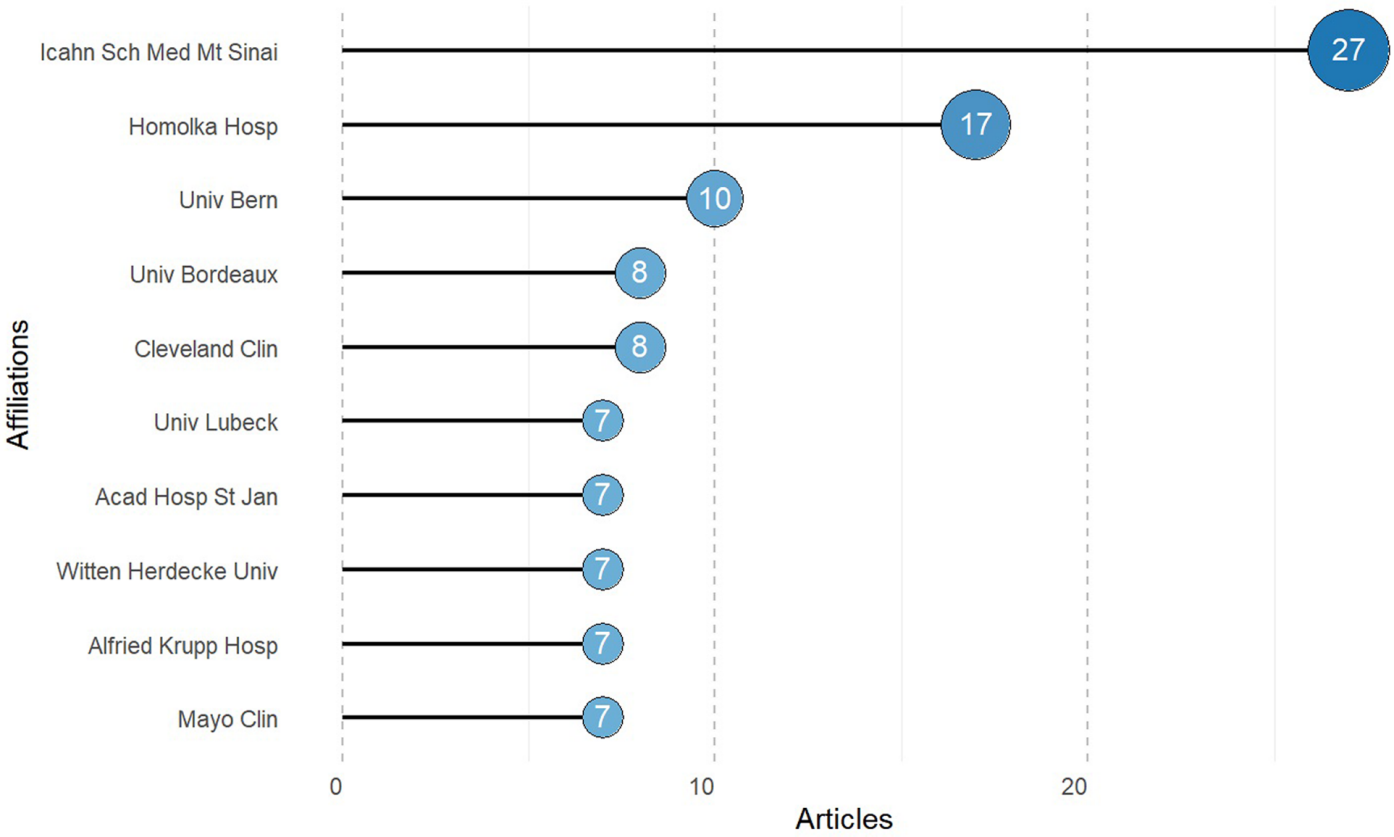
Top 10 institutions according to the number of publications.

In total, 435 institutions have been involved in research on the use of PFA in AF treatment; the top 10 are shown in Figure 4. The Icahn School of Medicine at Mount Sinai leads, with 27 publications, followed by Homolka Hospital with 17 publications, highlighting its substantial contributions to the field. Other notably active institutions include the University of Bern with 10 publications, Cleveland Clinic with 8, and the University of Bordeaux, also with 8 publications. These leading institutions are predominantly based in the United States and in European countries such as the Czech Republic, Switzerland, France, and Belgium.

The map of inter-institutional relationships generated with VOSviewer is presented as Supplementary Figure S1. The network comprises 136 nodes (representing different institutions), 5 clusters, and 811 links (indicating collaborations). Overall, the network is tightly connected, reflecting extensive cross-institutional cooperation in the field. The Icahn School of Medicine at Mount Sinai, the Texas Cardiac Arrhythmia Institute, and Mt. Sinai Fuster Heart Hospital occupy central positions, with high collaboration frequencies and numerous connections to other institutions. The Icahn School of Medicine at Mount Sinai is most central, underscoring its significant academic influence and leadership in the field. The time-overlapping co-authorship clustering network illustrates the patterns of collaboration among major institutions on the use of PFA for AF treatment and their evolving trends (Supplementary Figure S2). The Icahn School of Medicine at Mount Sinai and Na Homolce Hospital emerge as early pioneers in the field. Over time, the collaboration network has expanded, fostering more multinational and interdisciplinary research groups.

### Publication quantity and journal impact

In total, 52 journals have published articles on the use of PFA in AF treatment. The 10 journals with the largest number of publications are listed in Table 1. Leading journals include *Europace*, the *Journal of Interventional Cardiac Electrophysiology*, and the *Journal of Cardiovascular Electrophysiology*, which collectively account for 39% of the total publications. *Europace* leads with the largest number of articles (*n* = 39), representing 18% of all publications. Among the top 10 journals, *Circulation: Arrhythmia and Electrophysiology* has the greatest citation impact, with an average of 45 citations per article. Half of these journals are ranked in quartile 1 (Q1) of the Journal Citation Reports (JCR).

**Table 1:** Top 10 journals publishing the most articles on pulsed field ablation and atrial fibrillation.

Rank	Journals	Articles	Citations	Citation impact	IF	JCR-c
1	*Europace*	39	626	16	7.9	Q1
2	*Journal of interventional cardiac electrophysiology*	26	114	4	2.1	Q3
3	*Journal of cardiovascular electrophysiology*	20	217	11	2.3	Q3
4	*Jacc-clinical electrophysiology*	16	392	25	8.0	Q1
5	*Circulation-arrhythmia and electrophysiology*	14	635	45	9.1	Q1
6	*Heart rhythm*	12	249	21	5.6	Q1
7	*Frontiers in cardiovascular medicine*	10	62	6	2.8	Q2
8	*Journal of cardiovascular development and disease*	8	56	7	2.4	Q2
9	*Journal of clinical medicine*	8	51	6	3.0	Q1
10	*Pace-pacing and clinical electrophysiology*	7	15	2	1.7	Q3

IF, impact factor; JCR, journal citation reports.

### Author influences

In total, 1,119 authors have contributed to the development of this field (Table 2). V.Y. Reddy (30 publications, H-index = 19), P. Neuzil (22 publications, H-index = 14), and J. Petru (14 publications, H-index = 13) are the most influential of these authors, demonstrating the highest academic impacts in this area.

**Table 2:** Top 10 authors publishing the most articles on pulsed field ablation and atrial fibrillation.

Authors	Institution	Articles	H-index	citations	Citation impact
REDDY V.Y.	Icahn School of Medicine at Mount Sinai	30	19	1,670	56
NEUZIL P.	Homolka Hospital	22	14	1,272	58
PETRU J.	Homolka Hospital	14	13	1,141	82
SCHMIDT B.	MVZ CCB Frankfurt und Main-Taunus GbR	13	8	243	19
FUNASAKO M.	Homolka Hospital	11	11	722	66
KORUTH J.S.	Icahn School of Medicine at Mount Sinai	11	9	682	62
CHEN S.J.	Agaplesion Markus Hospital	10	6	177	18
DUKKIPATI S.R.	Icahn School of Medicine at Mount Sinai	10	10	1,047	105
JAIS P.	Bordeaux University	10	7	933	93
NATALE A.	Texas Cardiac Arrhythmia Institute	10	7	364	36
REICHLIN T.	University of Bern	10	7	101	10
VERMA A.	Southlake Regional Health Centre	10	8	410	41

The co-authorship clustering network is shown in Supplementary Figure S3. In total, 152 authors with three or more articles were grouped into seven clusters. Five clusters on the left of the figure form a tightly-knit collaborative community, reflecting frequent cooperation and strong academic connections among these researchers. In contrast, two clusters have few links to the others, indicating certain degrees of independence. The time-overlapping map for co-authorship highlights the rapid advancement of researchers such as Iacopino Saverio, Bianchi Stefano, and Malacrida Maurizio in this field (Supplementary Figure S4).

## Research hotspots

### Most cited publications

The 10 most frequently cited publications on the use of PFA in AF treatment, all of which have more than 100 citations, are listed in Supplementary Table S2. The most cited article, “Pulsed field ablation for pulmonary vein isolation in atrial fibrillation” (19) was published in 2019 and highlights the safety and efficacy of PFA; it has been cited more than 340 times. The second most cited article, “Pulsed field ablation of paroxysmal atrial fibrillation: 1-year outcomes of IMPULSE, PEFCAT, and PEFCAT II” (20) was published in 2021 in *JACC: Clinical Electrophysiology* and provides crucial clinical data on the outcomes and safety of PFA for AF treatment. These highly cited studies reflect the rapid development of and growing interest in PFA technology, reflecting its potential as an emerging approach for AF management.

### Citation bursts

Bursts of citation of the 25 most-cited publications in the field are illustrated in Supplementary Figure S5. The strongest citation burst is associated with the article by K. Neven et al. entitled “Acute and long-term effects of full-power electroporation ablation directly on the porcine esophagus”, published in 2017 in *Circulation: Arrhythmia and Electrophysiology* (20). This burst had a strength of 10.87 and lasted from 2019 to 2022. Another notable burst (strength = 9.51) is for the article by V.Y. Reddy et al. entitled “Ablation of atrial fibrillation with pulsed electric fields: an ultra-rapid, tissue-selective modality for cardiac ablation”, which was published in 2018 in *JACC: Clinical Electrophysiology* (21). Some recent citation bursts are ongoing, such as that for A. Hussein's et al. 2018 article entitled “Use of ablation index-guided ablation results in high rates of durable pulmonary vein isolation and freedom from arrhythmia in persistent atrial fibrillation patients: the PRAISE study results”, which began in 2021 (22). The article provides data on the effectiveness and safety of ablation index (AI)-guided pulmonary vein isolation (PVI) in patients with AF, showing that AI-guided ablation achieves high rates of durable isolation and reduces arrhythmia recurrence. It provides a comparative framework, theoretical support, and context for the use of PFA in AF treatment.

### Keyword frequency and clustering

**Figure 5:**
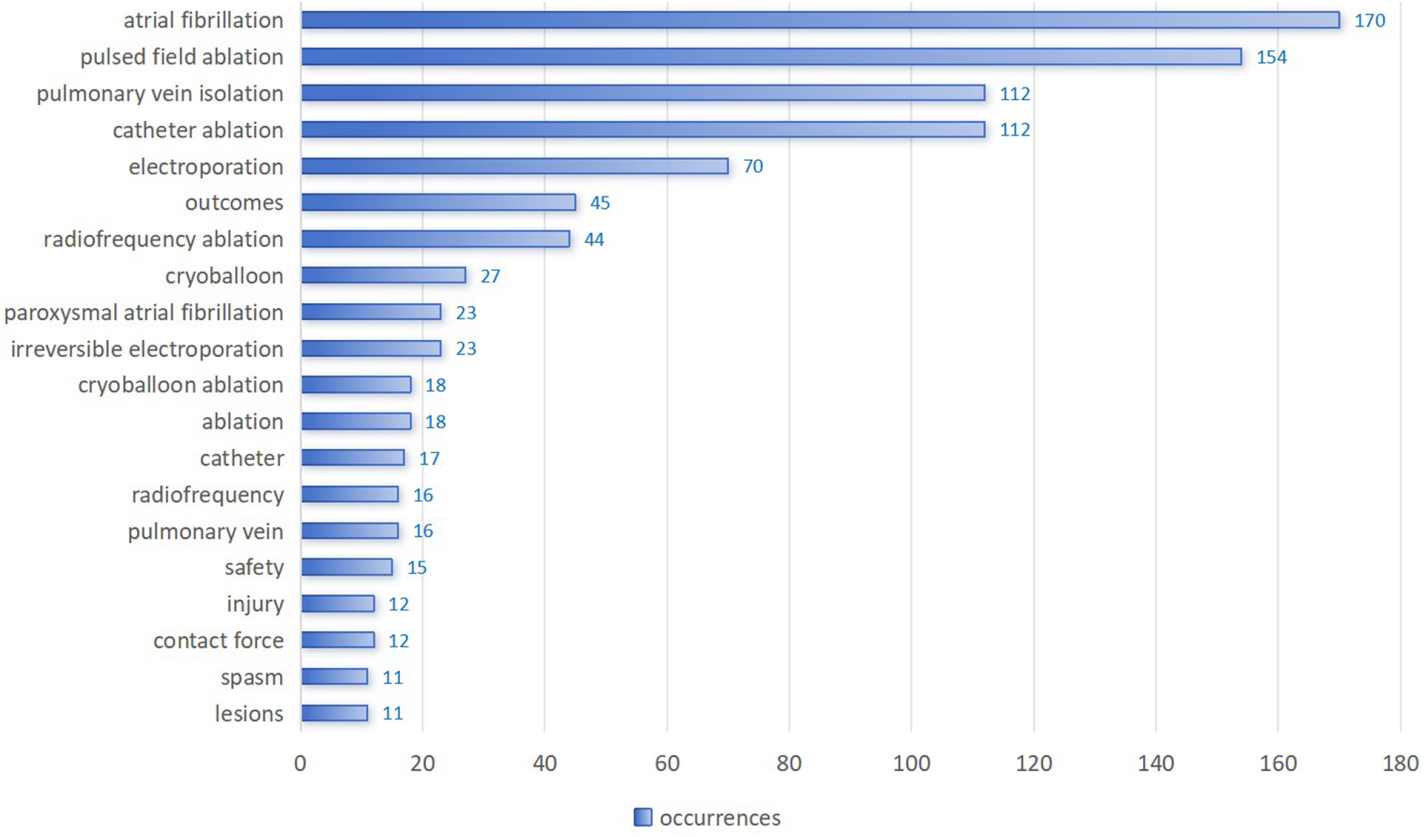
Top 20 most frequently used keywords.

**Figure 6:**
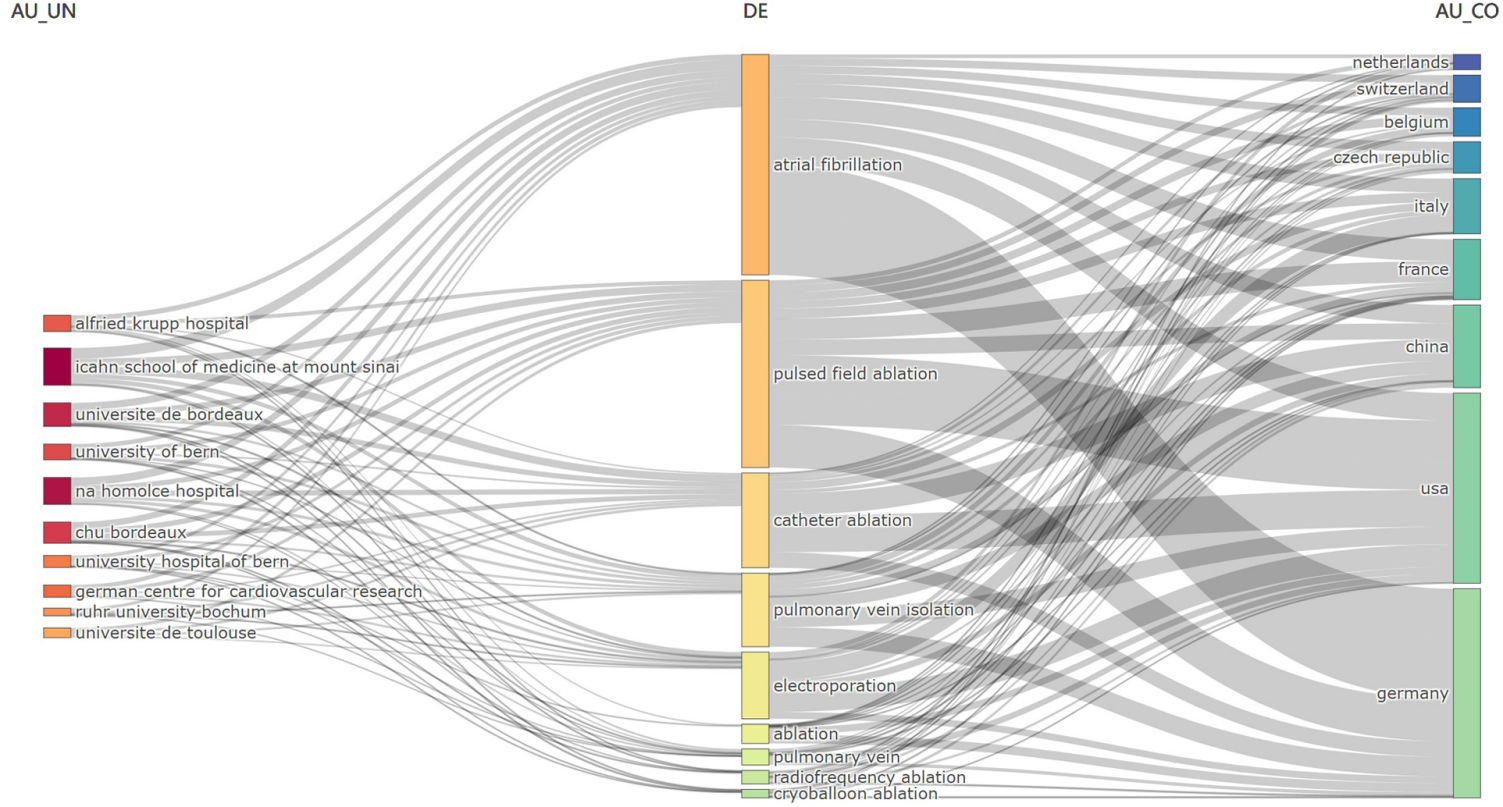
Three-field plot of keywords plus analysis of PFA and AF (left field: institutions; middle field: keywords; right field: countries).

Figure 5 shows the frequency of use of the top 20 of 586 identified keywords. “Atrial fibrillation” appeared most frequently, with 170 occurrences, followed by “pulsed field ablation” (154 occurrences) and “pulmonary vein isolation” (112 occurrences). Figure 6 illustrates the proportion of contribution of various institutions and countries to these core topics, showing associations and distribution. Nearly all institutions and countries have contributed to the nine topics represented by these keywords, although differences exist. For example, the Icahn School of Medicine at Mount Sinai has focused more on “atrial fibrillation” and “pulsed field ablation”, whereas Na Homolce Hospital has shown greater interest in “atrial fibrillation” and “electroporation”. The United States and Germany have made significant contributions across most of the key topics, whereas France and China have demonstrated more focused interest in “pulsed field ablation” and “electroporation”.

**Figure 7:**
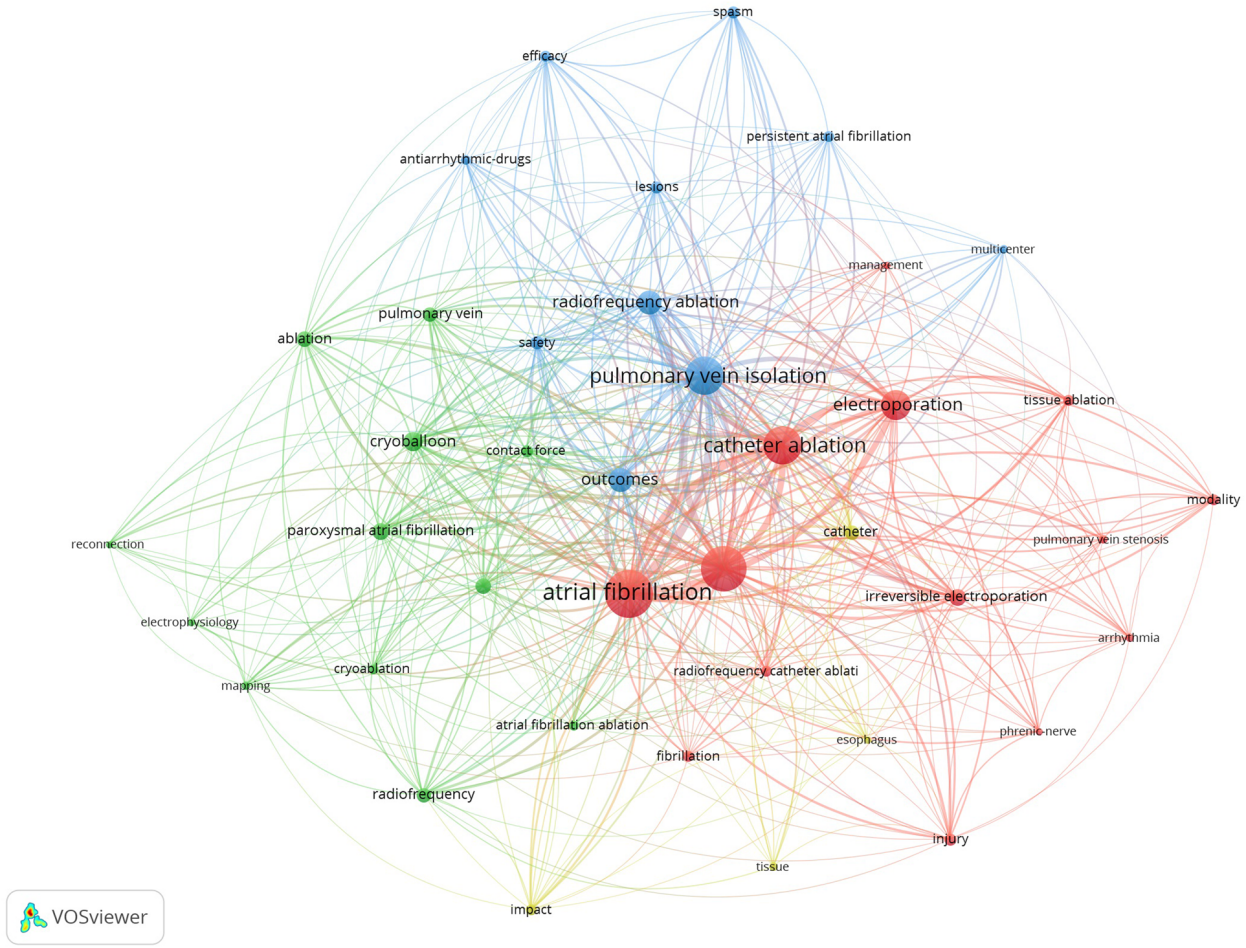
Keyword co-occurrence network. Each of the four clusters is represented by a different color. Node size reflects keyword use frequency and the distance between nodes reflect the strength of their relationships, with closely related keywords clustering together.

The co-occurrence network includes 40 keywords in four clusters (Figure 7). Cluster 1 (Figure 7, red) includes keywords related closely to PFA and AF, such as “arrhythmia”, “atrial fibrillation”, “catheter ablation”, “electroporation”, and “irreversible electroporation”. Cluster 2 (Figure 7, green) centers around ablation techniques, technical approaches, and their assessment and applications, with keywords including “cryoablation”, “cryoballoon”, “electrophysiology”, and “mapping”. Cluster 3 (Figure 7, blue) focuses on treatment outcomes, clinical effectiveness, and safety, including terms such as “antiarrhythmic drugs”, “efficacy”, “outcomes”, “safety”, “pulmonary vein isolation”, and “multicenter”. Cluster 4 (Figure 7, yellow) includes keywords such as “catheter”, “esophagus”, “impact”, and “tissue”, which are associated with potential effects on surrounding tissues and complications arising from ablation techniques. These clusters capture the diverse research areas in the field of PFA for AF.

**Figure 8:**
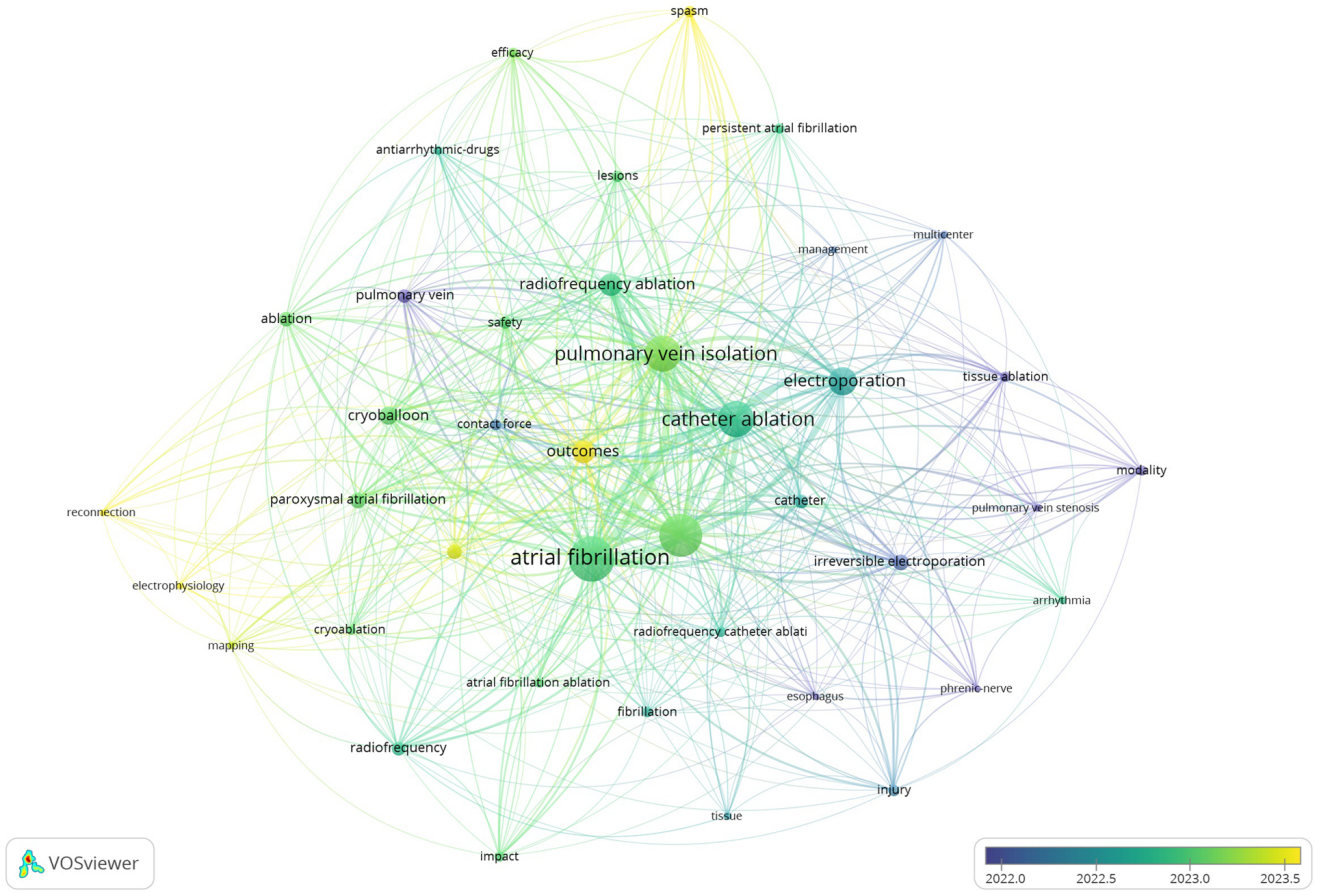
Time-overlapping keyword co-occurrence network. The color gradient from dark blue to light yellow indicates the average number of active years for the keywords.

The time-overlapping keyword co-occurrence network reflects the evolution of research foci over time (Figure 8). Early research is represented by terms such as “phrenic-nerve”, “tissue ablation”, “pulmonary vein stenosis”, and “modality”, reflecting a focus on the understanding of the techniques and complications of ablation therapies for AF. In contrast, more recent studies are represented by terms such as “spasm”, “reconnection”, “electrophysiology”, and “outcomes”, indicating a growing interest in the understanding of the mechanisms of ablation success and failure, improvement of techniques, and evaluation of the overall effectiveness and long-term results of ablation therapies applied in AF management. This shift highlights the ongoing evolution of the field, from foundational procedural knowledge to a deeper exploration of the optimization of patient outcomes and advancement of ablation technologies for AF treatment.

### Keyword citation bursts

**Figure 9:**
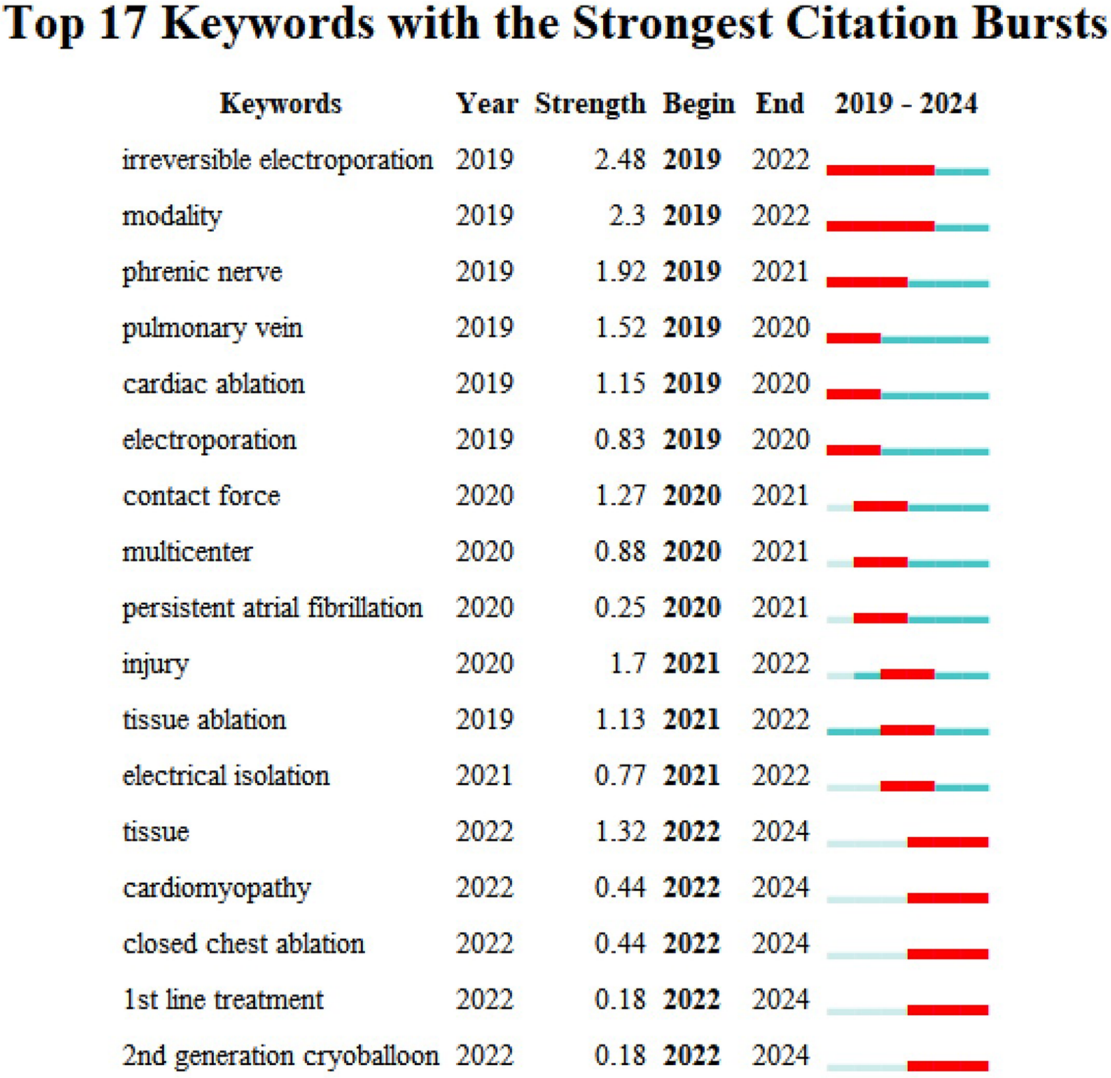
Top 25 keywords with strong citation bursts.

Figure 9 shows the 17 keywords with the strongest citation bursts. “Irreversible electroporation” and “modality” have burst from 2019 to 2022, reflecting a strong recent research interest in novel ablation techniques and their applications. “Phrenic nerve” (2019–2022), “pulmonary vein” (2019–2020), and “cardiac ablation” (2019–2020) also have notable citation bursts, highlighting their importance in the study of AF and related therapeutic approaches.

“Injury”, “tissue ablation”, and “electrical isolation” have bursts from 2021 to 2022, reflecting growing interest in the procedural outcomes and safety of new ablation methods. Emerging topics such as “tissue”, “cardiomyopathy”, “closed chest ablation”, “1st line therapy” and “2nd generation cryoballoon” have attracted considerable research attention since 2022, that may continue in coming years.

## Discussion

The growing body of literature on PFA for the treatment of AF reflects a dynamic and rapidly evolving field. Our comprehensive bibliometric analysis, covering 217 publications dating to 2019–2024, revealed key trends, leading contributors, and emerging research frontiers. This discussion synthesizes our findings, situates them within the broader context of cardiac electrophysiology, and explores future directions for the use of PFA in AF treatment.

The research on PFA for AF has progressed through two distinct phases. The stage extending from 2019 to 2021 was exploratory, characterized by a small but steadily increasing number of publications. This limited output likely reflects the nascent nature of PFA, with its concepts and applications still under validation and early clinical trials just beginning. From 2022 onward, PFA research expanded rapidly. This exponential growth signifies the increasing recognition of PFA's advantages in AF management, which generated heightened interest among researchers and clinicians. Factors contributing to this surge include the ability to selectively ablate myocardial tissue while minimizing damage to non-cardiac structures with PFA, which particularly reduces the risk of esophageal injury (23). However, it is also essential to consider the potential systemic effects of PFA, such as hemolysis. While PFA is known for its myocardial tissue selectivity, non-selective energy delivery can, in some cases, affect red blood cells, leading to hemolysis (24–26). Moreover, an increasing number of clinical trials and early research confirmed the efficacy and safety of PFA, solidifying its potential for clinical use (11, 26).

Many countries/regions have contributed to research on the use of PFA for AF, with the United States, Germany, and China leading. This result underscores these countries' pivotal roles in advancing research in this area. The collaboration network generated in this study highlights the central role of the United States in global research partnerships, possibly driven by its strong research infrastructure, substantial funding, and robust academic exchange platforms. Through these extensive international collaborations, the overall quality of research in this field is poised to advance significantly.

In total, 435 institutions involved in PFA and AF research produced publications, with the Icahn School of Medicine at Mount Sinai and Na Homolce Hospital leading the field. These institutions, along with others in the United States and Europe, have played key roles in advancing this research. The collaboration network generated in this study shows strong connections among 136 institutions. Central players, such as the Icahn School of Medicine and the Texas Cardiac Arrhythmia Institute, have high collaboration frequencies, reinforcing their influence. The evolving collaboration patterns reflect increasing multinational and interdisciplinary efforts in the field.

The distribution of articles across various journals underscores the academic influence of research on the use of PFA in AF treatment and highlights key platforms for knowledge dissemination. The top 10 journals in this field collectively represent a significant proportion of the research output. *Europace*, the *Journal of Interventional Cardiac Electrophysiology*, and the *Journal of Cardiovascular Electrophysiology* published 39% of all of the articles. Notably, *Circulation: Arrhythmia and Electrophysiology* had the largest citation impact (average, 45 citations/article) despite not having the largest publication volume. Half of the journals in the top 10 fall within the prestigious JCR Q1, indicating their strong influence and prominence in the field, which contributes significantly to shaping the landscape of research on PFA for AF treatment.

The most cited publications on the use of PFA for AF highlight significant advancements and growing recognition in the field. These influential studies emphasize PFA's safety and efficacy, marking it as a transformative approach in AF treatment. These works have become key references, shaping ongoing studies and clinical trials. As more data emerge, they will continue to guide the evolution of PFA technology, solidifying its role as a promising innovation in AF management. In 2019, Stewart et al. (27) conducted a preclinical study published in *Heart Rhythm* that validated the feasibility of PFA in animal models, demonstrating its superior safety and tissue specificity relative to traditional ablation methods. The study showed that PFA effectively induced targeted cardiomyocyte death, reduced electrogram amplitudes, and created lasting atrial lesions (27). The researchers reported that PFA lesions, unlike those created by RFA, had no thermal signature or residual cardiomyocytes and exhibited uniform fibrosis (27). They also demonstrated that PFA reduced epicardial fat inflammation and blood vessel remodeling, with no collateral damage to surrounding tissues (27). These findings highlight the ability to create precise, durable lesions with fewer inflammatory responses, positioning PFA as a safer alternative to traditional thermal-based ablation.

In the same year, Koruth et al. (28) published a study in *Circ-Arrhythmia Electrophysiology* that further supported these findings, reinforcing PFA's safety and efficacy as a cardiac ablation technique. They compared the use of PFA and traditional RFA for PVI, showing that PFA not only effectively ablates myocardial tissue but also largely spares surrounding non-cardiac structures (28). A key highlight of the study was the reporting of PFA's advantage in terms of preventing damage to critical areas such as the esophagus and phrenic nerve, a common concern with thermal ablation techniques (28). The non-thermal nature of PFA thus represented a significant breakthrough, minimizing procedural complications and enhancing patient safety. These preclinical studies laid the foundation for subsequent human trials, establishing PFA as a promising non-thermal ablation technique for AF.

Reddy et al. (19, 29–31) have comprehensively evaluated the efficacy and safety of PFA as an AF treatment, demonstrating its potential across various clinical contexts. In 2019, they published a groundbreaking study showing that rapid and durable PVI could be achieved with minimal damage to surrounding tissues and critical structures such as the esophagus and phrenic nerve, positioning PFA as a highly promising non-thermal technique and a safer alternative to RFA and cryoablation (19). The findings revealed that PFA can be used to selectively ablate myocardial tissue while avoiding damage to critical structures such as the esophagus and phrenic nerve. In 2020, Reddy et al. (30) reported on the extension of PFA's application to persistent AF cases by combining PVI with left atrial posterior wall ablation, with which they achieved excellent lesion durability and low complication rates, further validating PFA's potential in complex AF cases. A study published in 2021 provided 1-year follow-up data and reinforced these findings, confirming the maintenance of durable PVI in most patients and a significant reduction in AF recurrence, alleviating concerns about potential latent clinical issues due to the non-thermal mechanism of PFA (29). A 2023 study demonstrated that the efficacy of PFA was not inferior to that of conventional thermal ablation techniques (31). Overall, Reddy et al.'s research highlights the transformative potential of PFA for AF treatment. This technology enables safer and more efficient ablation with fewer adverse effects, and it is poised to become the new standard for AF ablation therapy as clinical validation continues.

A notable ongoing citation burst that began in 2021 was observed for Hussein et al.'s 2018 article entitled “Use of ablation index-guided ablation results in high rates of durable pulmonary vein isolation and freedom from arrhythmia in persistent atrial fibrillation patients: the PRAISE study results” (22). This sustained attention reflects the importance of the authors' finding that AI-guided PFA achieves high rates of durable PVI and long-term freedom from arrhythmia in persistent AF cases, offering valuable insights into the factors that contribute to successful AF ablation outcomes (22). PRAISE study citation is particularly relevant in PFA studies, as AI-guided RFA and PFA are applied with the aim of effectively creating lasting lesions. The study provides a comparative framework for PFA research, highlighting the significance of durable lesion formation for the minimization of AF recurrence.

The keyword co-occurrence and clustering analysis performed in this study provided an in-depth perspective on the evolving landscape of research on AF and PFA. The identification of four distinct clusters with different thematic concentrations underscores the complex, interdisciplinary nature of this field. Cluster 1, characterized by keywords such as “arrhythmia”, “atrial fibrillation”, and “irreversible electroporation”, reflects the foundational focus on the application of PFA within the broader context of AF management (32, 33). It highlights ongoing efforts to elucidate the mechanisms underlying electroporation, which distinguishes PFA from traditional thermal ablation techniques, offering a novel paradigm for tissue selectivity and safety. Cluster 2 emphasizes the technical aspects of ablation modalities (34, 35), with terms such as “cryoablation”, “cryoballoon”, and “mapping” pointing to comparative research on different catheter-based approaches. This cluster represents an area of intense investigation aimed at optimizing procedural outcomes, integrating advanced mapping techniques, and enhancing the precision of energy delivery to target tissues.

Cluster 3 focuses on treatment efficacy and safety, encompassing keywords such as “antiarrhythmic drugs”, “outcomes”, and “multicenter”. This cluster reflects increasing clinical evaluation of PFA and its long-term effectiveness (36, 37), as well as a broader emphasis on randomized controlled trials and multicenter studies to validate the technology's real-world applicability. The inclusion of terms related to clinical outcomes underscores the drive toward evidence-based assessment, with a particular emphasis on reducing atrial arrhythmia recurrence while minimizing complications. Cluster 4, which includes terms such as “esophagus”, “catheter”, and “impact”, centers on safety concerns associated with AF ablation, particularly the avoidance of collateral damage to surrounding tissues, such as the esophagus and phrenic nerve (23, 38). This research is crucial to address the major limitations of existing thermal ablation techniques, which have historically been associated with such complications, thereby further bolstering the case for the adoption of PFA due to its selective tissue effects and non-thermal mechanism of action.

The temporal evolution of keyword usage, revealed by the time-overlapping analysis, provides valuable insights into shifting priorities in AF ablation research. In the earlier phase, the focus was on the understanding of procedural challenges and mitigation of risks, as evidenced by the prevalence of terms such as “phrenic nerve”, “tissue ablation”, and “pulmonary vein stenosis”. This era was marked by the need to refine ablation techniques to address complications associated with thermal energy sources, such as esophageal injury and phrenic nerve damage (28, 39). The progression toward more recent terms such as “spasm”, “reconnection”, and “electrophysiology” reflects the maturation of the field, with the shifting of attention to the optimization of ablation efficacy and improvement of overall patient outcomes (26, 40). This shift mirrors a broader trend in cardiology and electrophysiology, with initial technological development giving way to a more sophisticated focus on clinical outcomes, durability, and procedural refinements. The increasing prominence of terms related to “outcomes” and “efficacy” further highlights the transition from a purely technical focus to a patient-centric approach, with the prioritization of long-term freedom from arrhythmia and improved quality of life (10, 41, 42). This ongoing evolution underscores the dynamic nature of AF ablation research, with a clear trajectory toward the enhancement of safety and efficacy, and the optimization of ablation technologies such as PFA to meet the clinical demands of diverse patient populations.

The identification of keywords with the strongest citation bursts provides insights into not only the current focal points of research on the use of PFA in AF treatment, but also potential future trajectories. It can guide researchers' and clinicians' focus for future studies and practices. Since 2019, the citation of terms such as “irreversible electroporation” and “modality” has increased substantially in this field, highlighting a marked interest in and the expanding application of innovative ablation technologies to improve clinical outcomes. Similarly, the increased focus on anatomical structures such as the “phrenic nerve” and “pulmonary vein” reflects a growing interest in ablation procedure safety and its enhancement. The increased citation of keywords such as “injury”, “tissue ablation”, and “electrical isolation” since 2021 also suggests mounting concern about the consequences and safety of ablation procedures, likely steering future research toward innovations that further minimize tissue damage and optimize isolation outcomes.

Topics emerging since 2022 are reflected by terms such as “tissue”, “cardiomyopathy”, “closed chest ablation”, “1st line therapy”, and “2nd generation cryoballoon” (34, 43, 44), and may become focal points in the future studies, particularly in the exploration of new treatment methods and their diverse clinical applications. Future research is expected to focus increasingly on the impact of pulsed ablation technology on cardiac tissues, with particular emphasis on the precise localization and minimization of the ablation area. Researchers will likely investigate ways in which to optimize the delivery of pulsed energy, thereby reducing collateral damage to surrounding healthy tissues while enhancing the accuracy and efficiency of targeted ablation. Moreover, optimizing contact force will be crucial to ensuring stable lesion formation and long-term durability, as variations in contact force can influence lesion depth and persistence, potentially affecting treatment outcomes (45, 46). Additionally, while PFA offers high tissue selectivity, risks such as circumflex artery spasm remain (47), particularly in areas near the coronary arteries, which could lead to myocardial injury. Future studies should aim to minimize these risks by improving energy delivery and utilizing real-time imaging guidance. It is also essential for future research to consider systemic effects, such as hemolysis, which may result from non-selective energy delivery, and to explore ways to optimize PFA's energy delivery to minimize such risks while maintaining its advantages in tissue selectivity.

This study has certain limitations. First, it was conducted only with English-language publications extracted from the WoSCC, which may have led to the exclusion of important research included in other databases and/or published in other languages, thereby limiting global representation. Second, due to citation delays and the rapidly evolving nature of PFA research, the full impacts of recent publications may not have been reflected in this study. Despite these limitations, the study provides useful insights into trends and emerging areas regarding the use of PFA for AF treatment.

## Conclusion

PFA is a transformative advancement in the treatment of AF, and this comprehensive bibliometric analysis highlights its rapid adoption and promising future in clinical electrophysiology. The continued evolution of PFA research will result in the further refinement and optimization of this modality to meet the complex needs of patients with AF, ensuring safer, more effective, and targeted ablation treatment.

## Data Availability

The raw data supporting the conclusions of this article will be made available by the authors, without undue reservation.
