# Supplementary material for: Pulsed field ablation for atrial fibrillation: a comprehensive bibliometric analysis of research trends and emerging Frontiers
Source: Front Cardiovasc Med. 2025 Feb 27;12:1513942. doi: 10.3389/fcvm.2025.1513942 (PMC11903418; doi:10.3389/fcvm.2025.1513942)
Supplement: Supplementary file 1 [file Datasheet1.pdf]

SUPPLEMENTARY MATERIAL

# Pulsed Field Ablation for Atrial Fibrillation: A Comprehensive Bibliometric Analysis of Research Trends and Emerging Frontiers

**Li Li[1], Bin Xie[2,*]**

[1]Chaozhou Central Hospital, Chaozhou, Guangdong, China.

[2]Department of Cardiovascular, The Second Affiliated Hospital of Shantou University Medical College, Shantou, Guangdong, China.

*** Correspondence:** Bin Xie 14bxie@stu.edu.cn

## Supplementary Figures and Tables

### *Supplementary Figures*

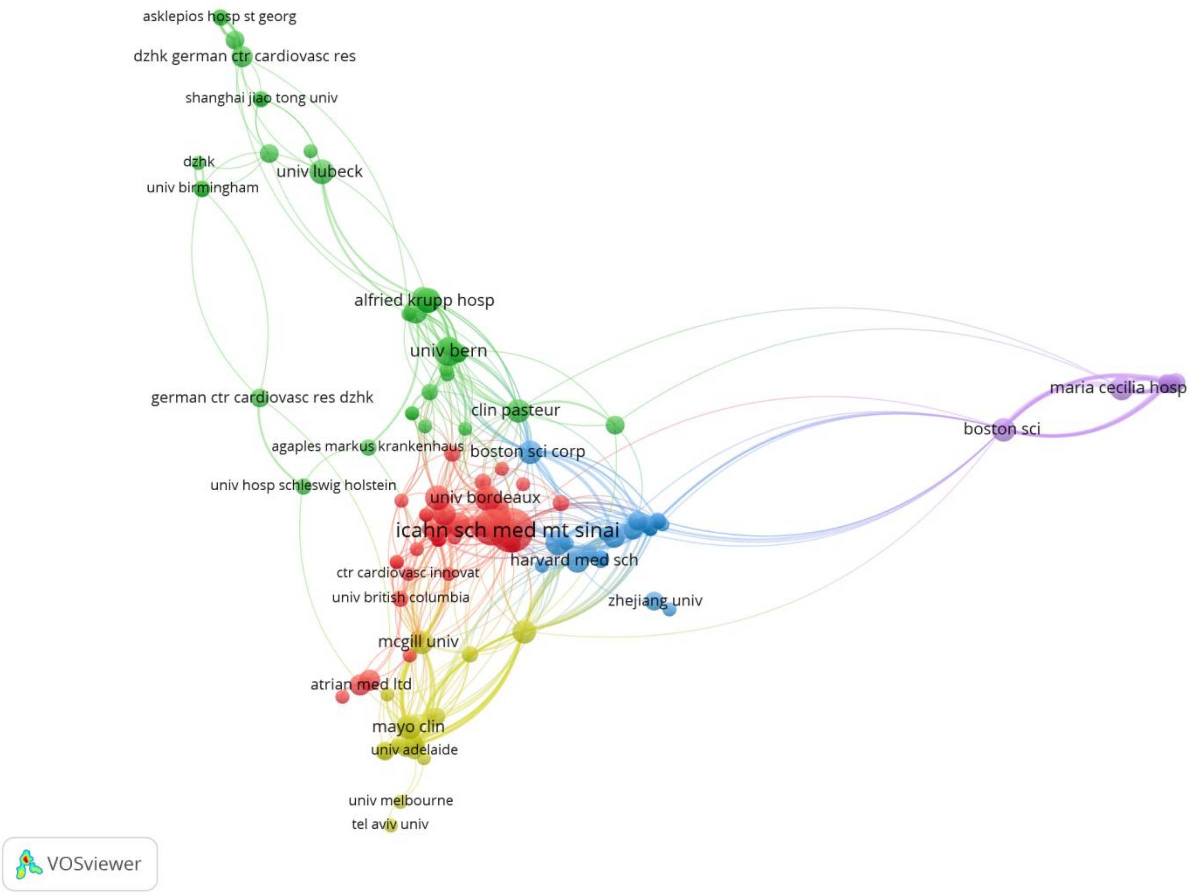


**Figure S1.** Clustering network of institutional co-authorship.

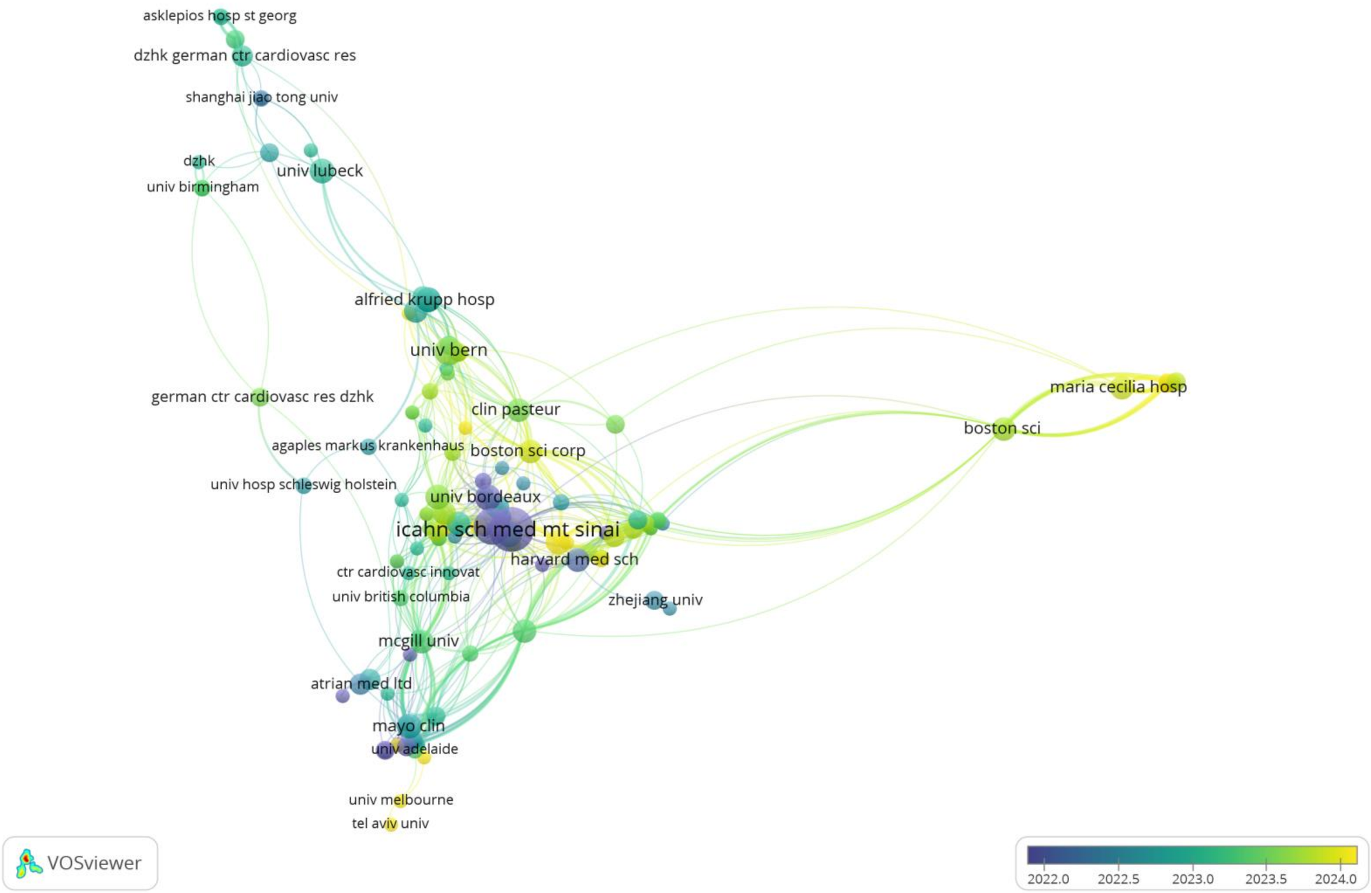


**Figure S2.** Time-overlapping institutional co-authorship network. The colors reflect institutions' average year of publication.

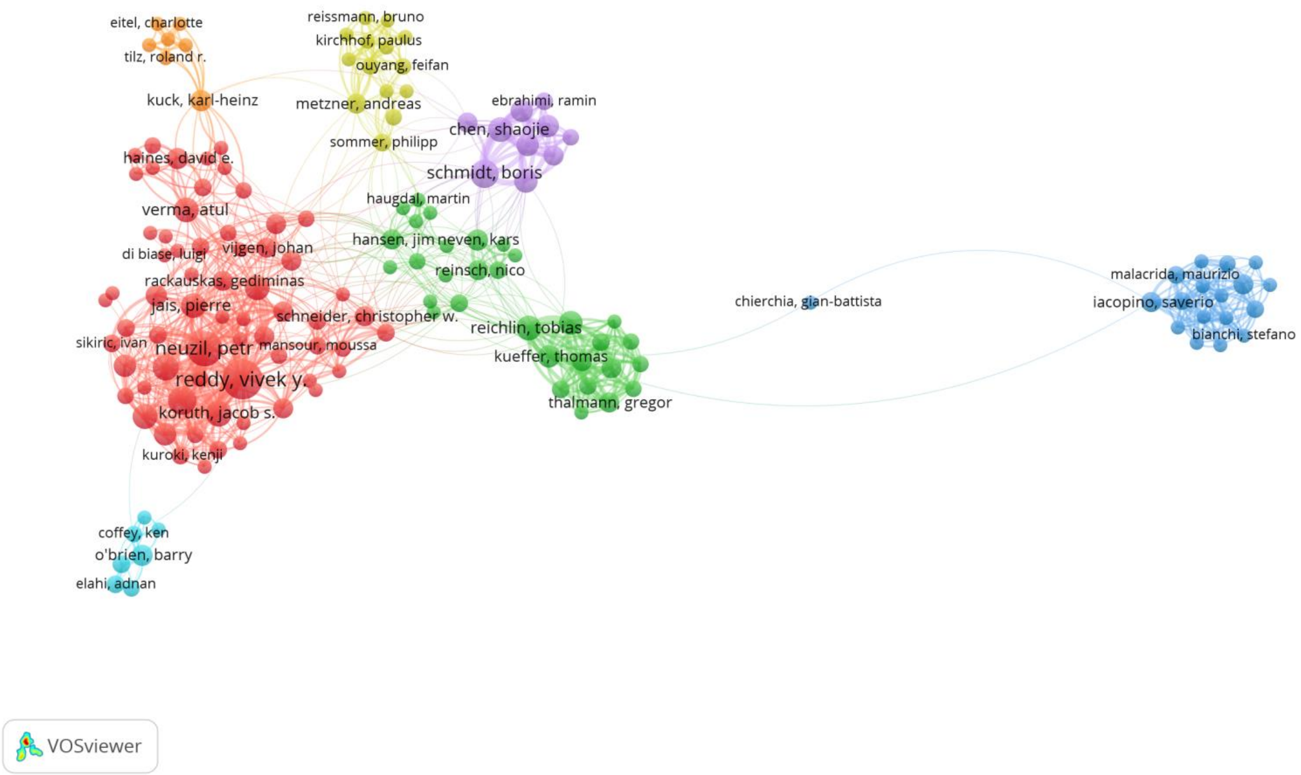


**Figure S3.** Clustering co-authorship network by authors. Each node represents an author; node size indicates the number of publications, the colors denote different collaboration clusters, and the lines represent the strength of collaborative ties.

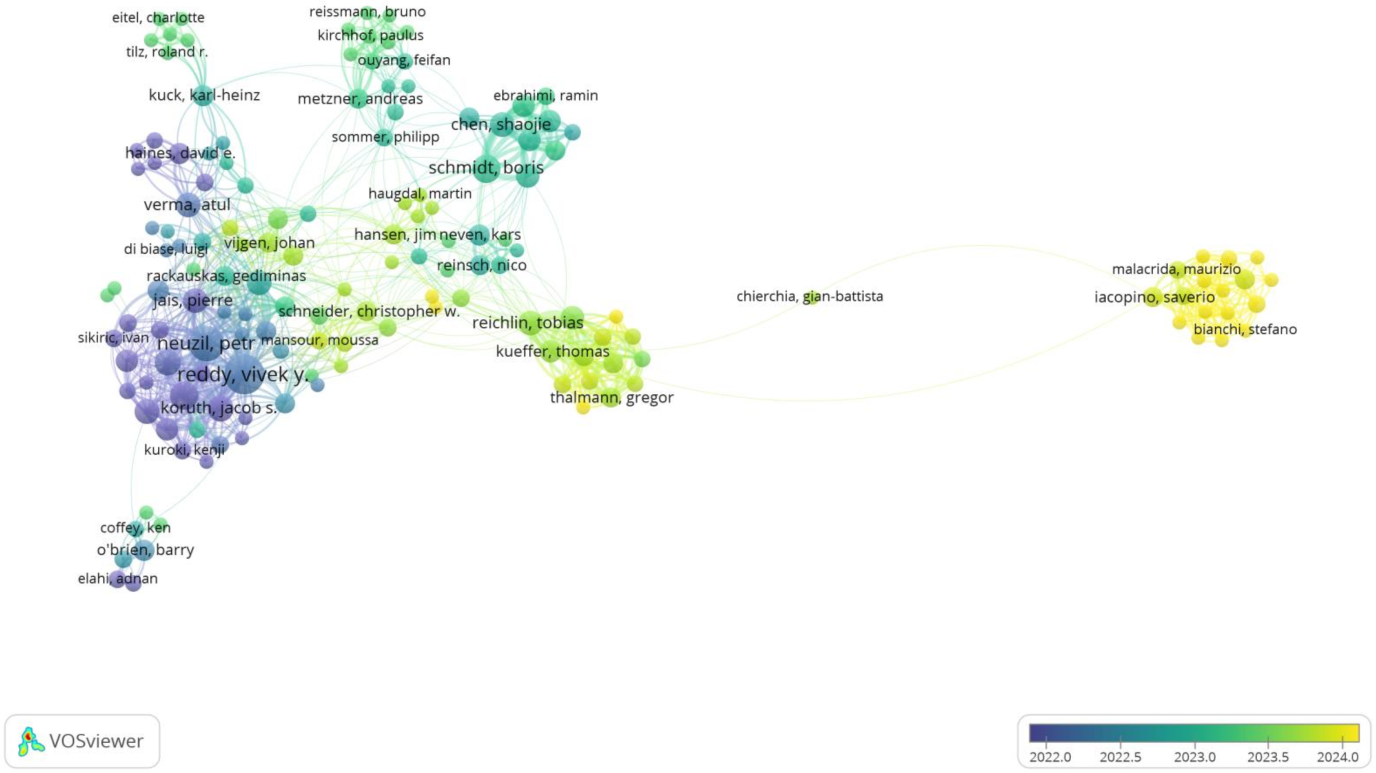


**Figure S4.** Time-overlapping co-authorship network by authors.

# Top 25 References with the Strongest Citation Bursts

| References | Year | Strength | Begin | End | 2019 - 2024 |
|---|---|---|---|---|---|
| Neven K, 2017, CIRC-ARRHYTHMIA ELEC, V10, P0, DOI 10.1161/CIRCEP.116.004672, DOI | 2017 | 10.87 | **2019** | 2022 | |
| Reddy VY, 2018, JACC-CLIN ELECTROPHY, V4, P987, DOI 10.1016/j.jacep.2018.04.005, DOI | 2018 | 9.51 | **2019** | 2022 | |
| van Driel VJHM, 2015, HEART RHYTHM, V12, P1838, DOI 10.1016/j.hrthm.2015.05.012, DOI | 2015 | 5.82 | **2019** | 2020 | |
| Stewart MT, 2019, HEART RHYTHM, V16, P754, DOI 10.1016/j.hrthm.2018.10.030, DOI | 2019 | 5.76 | **2019** | 2022 | |
| Calkins H, 2017, HEART RHYTHM, V14, PE275, DOI 10.1016/j.hrthm.2017.05.012, DOI | 2017 | 5.41 | **2019** | 2022 | |
| Kuck KH, 2016, NEW ENGL J MED, V374, P2235, DOI 10.1056/NEJMoa1602014, DOI | 2016 | 2.58 | **2019** | 2020 | |
| Wojtaszczyk A, 2018, J CARDIOVASC ELECTR, V29, P643, DOI 10.1111/jce.13454, DOI | 2018 | 2.45 | **2019** | 2022 | |
| Calkins H, 2017, HEART RHYTHM, V14, PE445, DOI 10.1016/j.hrthm.2017.07.009, DOI | 2017 | 2.43 | **2019** | 2021 | |
| Koruth J, 2019, CIRC-ARRHYTHMIA ELEC, V12, P0, DOI 10.1161/CIRCEP.119.007781, DOI | 2019 | 6.88 | **2020** | 2022 | |
| Koruth JS, 2020, EUROPACE, V22, P434, DOI 10.1093/europace/euz341, DOI | 2020 | 4.34 | **2020** | 2021 | |
| Muthalaly RG, 2018, J CARDIOVASC ELECTR, V29, P854, DOI 10.1111/jce.13484, DOI | 2018 | 3 | **2020** | 2022 | |
| Witt CM, 2018, J AM HEART ASSOC, V7, P0, DOI 10.1161/JAHA.118.009575, DOI | 2018 | 2.85 | **2020** | 2021 | |
| Sugrue A, 2019, J INTERV CARD ELECTR, V55, P251, DOI 10.1007/s10840-019-00574-3, DOI | 2019 | 2.44 | **2020** | 2022 | |
| Reddy VY, 2020, CIRC-ARRHYTHMIA ELEC, V13, P0, DOI 10.1161/CIRCEP.120.008718, DOI | 2020 | 2.13 | **2020** | 2022 | |
| Reddy VY, 2019, J AM COLL CARDIOL, V74, P315, DOI 10.1016/j.jacc.2019.04.021, DOI | 2019 | 1.96 | **2020** | 2021 | |
| Iwasawa J, 2017, J AM COLL CARDIOL, V70, P542, DOI 10.1016/j.jacc.2017.06.008, DOI | 2017 | 1.85 | **2020** | 2021 | |
| Loh P, 2020, CIRC-ARRHYTHMIA ELEC, V13, P1083, DOI 10.1161/CIRCEP.119.008192, DOI | 2020 | 3.57 | **2021** | 2022 | |
| Howard B, 2020, CIRC-ARRHYTHMIA ELEC, V13, P0, DOI 10.1161/CIRCEP.120.008337, DOI | 2020 | 2.88 | **2021** | 2022 | |
| Padmanabhan D, 2019, J CARDIOVASC ELECTR, V30, P607, DOI 10.1111/jce.13860, DOI | 2019 | 2.63 | **2021** | 2022 | |
| Koruth JS, 2020, CIRC-ARRHYTHMIA ELEC, V13, P0, DOI 10.1161/CIRCEP.119.008303, DOI | 2020 | 2.42 | **2021** | 2022 | |
| Wittkampf FHM, 2018, JACC-CLIN ELECTROPHY, V4, P977, DOI 10.1016/j.jacep.2018.06.005, DOI | 2018 | 2.24 | **2021** | 2022 | |
| Kuroki K, 2020, HEART RHYTHM, V17, P1528, DOI 10.1016/j.hrthm.2020.04.040, DOI | 2020 | 2.18 | **2021** | 2022 | |
| Avazzadeh S, 2020, J CLIN MED, V9, P0, DOI 10.3390/jcm9103081, DOI | 2020 | 2.16 | **2021** | 2022 | |
| van Es R, 2019, BIOMED ENG ONLINE, V18, P0, DOI 10.1186/s12938-019-0693-7, DOI | 2019 | 2.16 | **2021** | 2022 | |
| Hussein A, 2018, CIRC-ARRHYTHMIA ELEC, V11, P0, DOI 10.1161/CIRCEP.118.006576, DOI | 2018 | 1.46 | **2021** | 2024 | |

**Figure S5.** Top 25 publications on PFA and AF with the strongest citation bursts. The blue line represents the observation period (2019–2024) and the red lines indicate burst durations.

***Supplementary Tables***

**Table S1.** Top 10 countries with high frequencies of collaborative publications.

| From | To | Frequency |
|---|---|---|
| USA | CZECH REPUBLIC | 31 |
| USA | FRANCE | 19 |
| FRANCE | CZECH REPUBLIC | 15 |
| USA | BELGIUM | 15 |
| USA | CANADA | 15 |
| GERMANY | NETHERLANDS | 14 |
| GERMANY | USA | 14 |
| BELGIUM | AUSTRIA | 13 |
| FRANCE | BELGIUM | 13 |
| USA | NETHERLANDS | 13 |

**Table S2.** The top 10 cited publications.

| Rank | Title | Year, Journal | First author | Total Citations | TC per Year |
|---|---|---|---|---|---|
| 1 | Pulsed Field Ablation for Pulmonary Vein Isolation in Atrial Fibrillation | 2019, J AM COLL CARDIOL | REDDY VY | 340 | 56.67 |
| 2 | Pulsed Field Ablation of Paroxysmal Atrial Fibrillation: 1-Year Outcomes of IMPULSE, PEFCAT, and PEFCAT II | 2021, JACC-CLIN ELECTROPHY | REDDY VY | 239 | 59.75 |
| 3 | Pulsed Field Ablation in Patients With Persistent Atrial Fibrillation | 2020, J AM COLL CARDIOL | REDDY VY | 190 | 38.00 |
| 4 | Multi-national survey on the methods, efficacy, and safety on the post-approval clinical use of pulsed field ablation (MANIFEST-PF) | 2022, EUROPACE | EKANEM E | 156 | 52.00 |
| 5 | Pulsed Field Ablation for the Treatment of Atrial Fibrillation: PULSED AF Pivotal Trial | 2023, CIRCULATION | VERMA A | 139 | 69.50 |
| 6 | Intracardiac pulsed field ablation: Proof of feasibility in a chronic porcine model | 2019, HEART RHYTHM | STEWART MT | 130 | 21.67 |
| 7 | Pulsed Field or Conventional Thermal Ablation for Paroxysmal Atrial Fibrillation | 2023, NEW ENGL J MED | REDDY VY | 121 | 60.50 |
| 8 | Preclinical Evaluation of Pulsed Field Ablation: Electrophysiological and Histological Assessment of Thoracic Vein Isolation | 2019, CIRC-ARRHYTHMIA ELEC | KORUTH J | 115 | 19.17 |
| 9 | Pulsed field ablation selectively spares the oesophagus during pulmonary vein isolation for atrial fibrillation | 2021, EUROPACE | COCHET H | 102 | 25.50 |

| | | | | | |
|---|---|---|---|---|---|
| 10 | Lattice-Tip Focal Ablation Catheter That Toggles Between Radiofrequency and Pulsed Field Energy to Treat Atrial Fibrillation: A First-in-Human Trial | 2020, CIRC-ARRHYTHMIA ELEC | REDDY VY | 102 | 20.40 |

Abbreviations:TC, total citations.
